# Targeted DNA and RNA sequencing of fine-needle biopsy FFPE specimens in patients with unresectable hepatocellular carcinoma treated with sorafenib

**DOI:** 10.18632/oncotarget.4270

**Published:** 2015-05-25

**Authors:** Kazuko Sakai, Haruhiko Takeda, Norihiro Nishijima, Etsuro Orito, Kouji Joko, Yasushi Uchida, Namiki Izumi, Kazuto Nishio, Yukio Osaki

**Affiliations:** ^1^ Department of Genome Biology, Kinki University Faculty of Medicine, Ohono-Higashi, Osaka-Sayamashi, Osaka, Japan; ^2^ Department of Gastroenterology and Hepatology, Osaka Red Cross Hospital, Osaka, Japan; ^3^ Department of Gastroenterology and Hepatology, Nagoya Daini Red Cross Hospital, Nagoya City, Japan; ^4^ Center for Liver-Biliary-Pancreatic Diseases, Matsuyama Red Cross Hospital, Matsuyama City, Japan; ^5^ Department of Gastroenterology, Matsue Red Cross Hospital, Matsue City, Japan; ^6^ Department of Gastroenterology and Hepatology, Musashino Red Cross Hospital, Musashino, Japan; ^7^ Department of Gastroenterology and Hepatology, Graduate School of Medicine, Kyoto University, Shogoin-Kawaharacho, Kyoto, Japan

**Keywords:** hepatocellular cancer, sorafenib, response, mutation

## Abstract

The multi-kinase inhibitor sorafenib is now used as standard therapy for advanced hepatocellular carcinoma (HCC). Predictive biomarkers of response to sorafenib are thus necessary. The purpose of this study was to assess the feasibility of using targeted DNA and RNA sequencing to elucidate candidate biomarkers of sorafenib response using fine-needle biopsy, formalin-fixed paraffin-embedded (FFPE) specimens in patients with HCC. Targeted DNA and RNA deep sequencing were feasible for the evaluation of fine-needle biopsy FFPE specimens obtained from 46 patients with HCC treated with sorafenib. Frequent mutations of suppressor genes, such as *CTNNB1* (34.8%) and *TP53* (26.1%), were detected in the HCC tumors. After excluding these suppressor genes, the average numbers of detected oncogene mutations differed significantly between the non-PD and PD groups (*P* = 0.0446). This result suggests that the oncogene mutational burden in the tumor might be associated with the clinical response to sorafenib. We have identified candidate gene expression (*TGFa, PECAM1*, and *NRG1*) in tumor for the prediction of sorafenib response and PFS by RNA sequencing. Our findings provide new insights into biomarkers for sorafenib therapy and allow us to discuss future therapeutic strategies.

## INTRODUCTION

Hepatocellular carcinoma (HCC) is a common malignancy worldwide, and the at-risk population is still growing [1, 2]. Several reports have suggested that hepatocarcinogenesis involves multiple molecular pathways and the accumulation of genetic and epigenetic alterations, including copy number aberrations and gene mutations [3, 4]

The multi-kinase inhibitor sorafenib is now used as standard therapy for advanced HCC. In two pivotal clinical studies, a response was observed in 3.3% (5/150) and 0.7% (2/299) of patients treated with sorafenib [5, 6]. Despite its low response rate, we occasionally encounter HCC patients with good response to sorafenib, and their clinical characteristics have been recently investigated in Japan [7]. Complete responses were also observed in patients with unresectable HCC after short-term treatment with sorafenib [8-10]. In a retrospective study, we previously analyzed the clinical and molecular backgrounds of 13 responders to sorafenib who experienced significant tumor shrinkage [11]. A comparative genomic hybridization analysis using one frozen HCC sample from a responder demonstrated that the 11q13 region, a rare amplicon in HCC including the loci for *FGF3* and *FGF4*, was highly amplified. A real-time polymerase chain reaction-based copy number assay revealed that *FGF3/FGF4* amplification was observed in 3 of the 10 HCC samples from responders with evaluable DNA samples. *FGF3/FGF4* amplification is thus considered to be a possible mechanism involved in the response to sorafenib. However, the mechanisms responsible for the response to sorafenib in the remaining seven cases remain unclear. Here, we conducted a prospective and retrospective study to elucidate the other mechanisms related to sorafenib response and to find out the predictive biomarkers for sorafenib response.

Recent cancer profiling studies have focused on next-generation sequencing (NGS). The development of HCC is a multistep process that involves the accumulation of a wide range of genetic and phenotypic alterations, leading to the aberrant expression of genes that regulate cell proliferation. Therefore, somatic mutations are often detected in HCC [12, 13], and these mutations are part of key mechanisms resulting in carcinogenesis. Somatic mutations of cancer-related genes are also related to the sensitivity and resistance of solid tumors to targeted drugs. For example, the *KRAS* mutation status in colorectal cancer is related to the effectiveness of anti-epidermal growth factor receptor (EGFR) antibodies [14].

The use of targeted DNA and RNA sequencing using NGS technology has been limited to clinical formalin-fixed paraffin-embedded (FFPE) samples. We have focused on molecular profiling using FFPE samples [15, 16]. However, few previous studies have compared the mutation profiles of HCC biopsy samples and the response to sorafenib treatment.

RNA sequencing of steady-state RNA expression avoids the limitations of microarray expression and allows for the massive parallel sequencing of millions of sequences on chips containing complementary DNA (cDNA) libraries, generating a higher number of transcript sequences than is possible using a microarray analysis [17]. Thus, RNA sequencing presents unprecedented possibilities for genomic characterization. The expression data for more than 20,000 genes is thought to have a high redundancy, and a large sample size is necessary for validation. Therefore, for this study, we used a multiple gene expression analysis for FFPE samples using targeted RNA sequencing as well as DNA sequencing. This gene set was selected for targeted genes or genes related to drug sensitivity.

## RESULTS

### Patients and sample collection

In this study, we assessed 46 specimens obtained from HCC patients treated with sorafenib. The characteristics of the patients are summarized in Table 1. Twenty-four patients were non-PD group (PR + SD) and 18 patients were PD group to sorafenib, as determined using the RECIST criteria. Response was not evaluable (NE) in four patients. Most of the specimens (40/46, 87.0%) were obtained by liver biopsy. The 46 specimens were subjected to DNA and RNA extraction, yielding median amounts of 266.9 ng (range, 53.2 to 474.0) and 97.7 ng (range, 3.2 to 6555.6), respectively. The results of the DNA and RNA extraction are shown in Supplementary Table S1.

**Table 1:** Patient characteristics (n = 46)

		No. (%)
Age, years	Median (range)	73.5 (45-86)
	< 65	14 (30.4)
	≧ 65	32 (69.6)
Sex	Male	34 (73.9)
	Female	12 (26.1)
Stage	II	2 (4.3)
	III	16 (34.8)
	IVA	12 (26.1)
	IVB	16 (34.8)
Histology	Well differentiated	17 (37.0)
	Moderate differentiated	16 (34.8)
	Poorly differentiated	9 (19.6)
	Undifferentiated	4 (8.7)
Tissue	Liver biopsy specimen	40 (87.0)
	Surgical specimen	6 (13.0)
Response to sorafenib	PR	2 (4.3)
(RECIST)	SD	22 (47.8)
	PD	18 (39.1)
	NE	4 (8.7)

PR, partial response; SD, stable disease; PD, progressive disease; NE, not evaluable

### Analysis of somatic mutations

Somatic hotspot mutations in 50 genes were screened using the Ion AmpliSeq Cancer Hotspot Panel v2. Mutation profiling was successfully performed in all the cases. The average read number of all the samples was 472,016 (range, 101,044 to 1,139,936). The average read number of amplicons was 2,280 (range, 22 to 30,083), allowing the detection of mutations in samples containing approximately 1% tumor cells.

**Figure 1:**
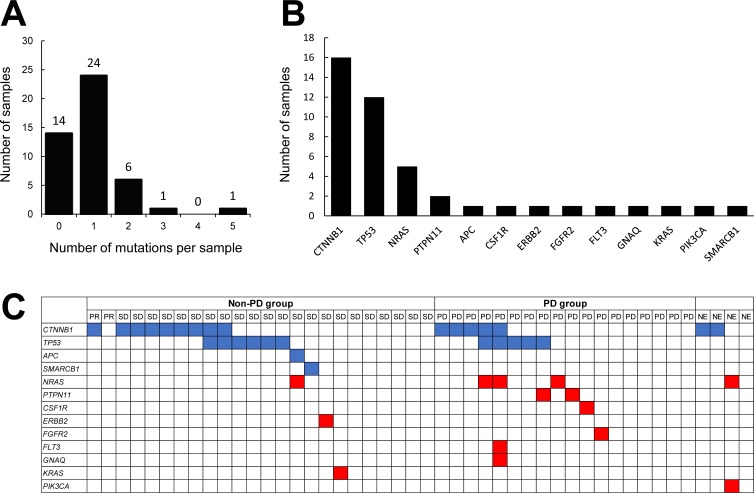
Analysis of somatic gene mutations in FFPE specimens obtained from HCC patients **A.** Mutations in 50 targeted genes were detected in 46 specimens using DNA sequencing. The number of mutations per sample is depicted along the x-axis, and the number of samples is shown on the y-axis. **B.** Mutated genes in HCC tumors. The x-axis shows the symbols for the mutated genes. The y-axis represents the total number of mutation events detected among the 46 samples. **C.** Distribution of all types of mutations detected in the samples. The top row represents the 46 HCC cases categorized according to their response to sorafenib. The rows beneath represent individual gene mutations (blue, tumor suppressor genes; red, oncogenes).

A somatic mutation in at least one gene was identified in 32 of the 46 specimens (69.9%). Six specimens (13.0%) were positive for mutations in two genes, one specimen had mutations in three genes, and one specimen had mutations in five genes (Figure 1A). We identified *CTNNB1* mutations in 16 patients (34.8%), *TP53* mutations in 12 (26.1%), *NRAS* mutations in 5 (10.9%), *PTPN11* mutations in 2 (4.3%), and *APC, CSF1R, ERBB2, FGFR2, FLT3, GNAQ, KRAS, PIK3CA*, and *SMARCB1* mutations in 1 each (2.2%) (Figure 1B). All the mutational statuses are shown in Supplementary Table S2. This mutation profile for HCC tumors was consistent with those of previous reports [12].

To explore differences between the non-PD and PD groups, we compared the number of mutations. Among the 46 patients, 24 were classified as non-PD group (PR + SD) and 18 were classified as PD group, according to the RECIST criteria. The 4 NE patients were excluded from the analysis. Among the 24 non-PD group, 14 specimens were positive for mutations in one gene and three specimens had mutations in two genes (Figure 1C). Among the 18 PD group, nine specimens were positive for mutations in one gene, one specimen had mutations in two genes, one specimen had mutations in three genes, and one specimen had mutations in five genes. The average numbers of mutations in the non-PD and PD groups were 0.83 and 1.05, respectively (Table 2). These values were not significantly different. Next, we excluded the mutations in tumor suppressor genes (*CTNNB1, TP53, APC*, and *SMARCB1*) and compared the number of mutations in non-PD and in non-PD groups. Among the 24 non-PD group, only three specimens were positive for mutations in one gene. Among the 18 PD group, six specimens were positive for mutations in one gene and one specimen had mutations in three genes (Figure 1C). The average numbers of mutations in the non-PD and PD groups were 0.13 and 0.50, respectively, and these values were significantly different (*P* = 0.0446, Chi-squared test) (Table 2). This result suggests that the oncogene mutational burden in the tumor might be associated with the clinical response to sorafenib.

**Table 2:** Relationship between gene mutation and clinical response to sorafenib

		All patients (n = 46) No. (%)	Average number of mutations per sample	P*	Average number of mutations per sample (Exclusion of tumor suppressor genes ^a)^)	P*
Response to sorafenib	Non-PD	24 (52.2)	0.88	.554	0.13	.045*
(RECIST)	PD	18 (39.1)	1.06		0.50	
	NE	4 (8.7)	-		-	

* *P* < 0.05 (Chi-squared test)

a) *APC, CTNNB1, SMARCB1*, and *TP53* were excluded

**Figure 2:**
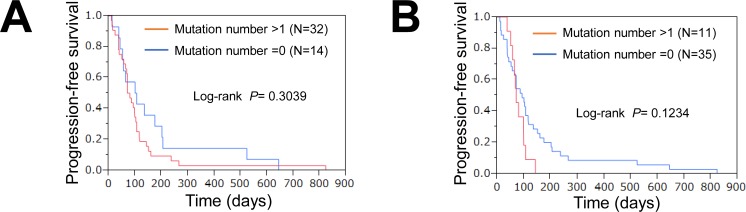
Association of mutations with response or survival **A.** PFS curves for patients with and those without gene mutations. Red, positive for one or more mutations; blue, no mutations. **B.** PFS curves for patients with and those without oncogenes. Mutations in tumor suppressor genes were excluded. Red, positive for one or more mutations; blue, no mutations.

The 46 patients were classified into mutation-positive (one or more mutations) and mutation-negative (no mutations) groups. The survival curves for these two groups are shown in Figure 2A. The median progression-free survival (PFS) periods of the patients with or without gene mutations were 103 and 76 days, respectively (*P* = 0.3039, log-rank test). The median PFS of the patients with or without gene mutations, excluding suppressor genes, were 93 and 71 days (*P* = 0.1234, log-rank test), respectively (Figure 2B). The mutation-negative group tended to have a longer PFS, but the difference was not statistically significant.

### Analysis of gene expression

**Figure 3:**
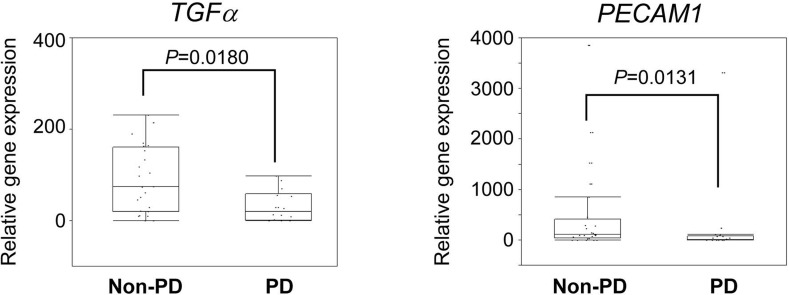
Association of gene expression profiles with response The expression levels of *TGF*α and *PECAM1* in non-PD and PD groups are shown. Relative expression (raw read number/total read number) of each gene was calculated. The median value is indicated by the horizontal bar on the graphs (Man-Whitney U-test for *P* values).

The gene expression analysis was performed using targeted RNA sequencing. Fifty target genes, consisting of receptor tyrosine kinase and its ligands, were selected for RNA sequencing. The median read number for the 46 samples was 188,213 (range, 1,628 to 319,039). A coverage of over 100,000 was achieved in 89.1% of the RNA samples (41/46). The data from RNA sequencing was normalized according to each read number. We then compared the relative gene expressions between non-PD and PD groups. The 4 NE patients were excluded from the analysis. The expressions of *TGFa* (median, 74.1 *vs*. 20.3, *P* = 0.0180) and *PECAM1* (median, 110.2 *vs*. 13.2, *P* = 0.0131) were significantly increased in the non-PD group (Figure 3). No other significant associations were observed between the gene expression levels and the PFS (Supplementary Table S3).

**Figure 4:**
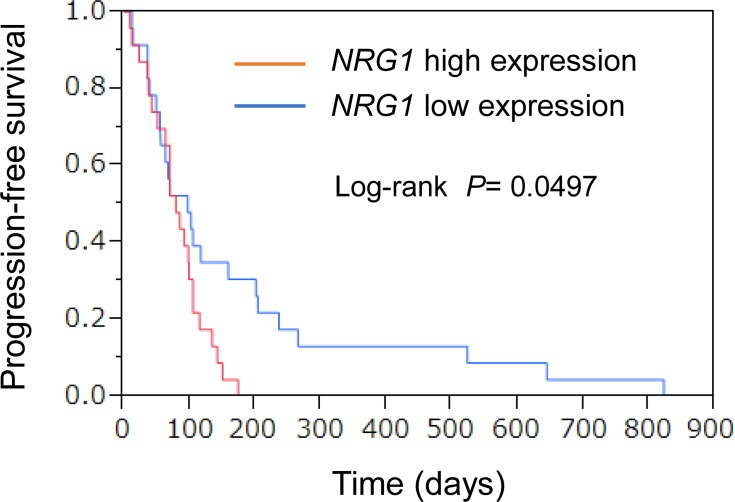
Association of gene expression profiles with PFS Relative expression (raw read number/total read number) of each gene was calculated. Kaplan-Meier curves for univariate analyses (log-rank) for patients with low *NRG1* expression (blue) versus high *NRG1* expression (red) tumors.

The median values for each gene were defined as the cutoff values separating the high and low gene expression groups. The PFS of the patients with a low *NRG1* expression was longer than that of the patients with a high *NRG1* expression (Figure 4). The median PFS periods of the patients with low and high *NRG1* expressions were 98 and 80 days, respectively (*P* = 0.0497, log-rank test). The expression of other genes, including *TGFa* and *PECAM1*, were not associated with PFS (Supplementary Table S4). These results suggest that the *NRG1* expression level in the tumor tissue might be a predictor of a good PFS.

## DISCUSSION

In the present study, we tested the use of FFPE specimens of HCC tissue for DNA and RNA sequencing using NGS technology. Most of the collected specimens obtained by liver biopsy were small in size. DNA and RNA were extracted at the same time from 10 slices of one biopsy sample. The yields of DNA and RNA were sufficient for the DNA and RNA sequencing, and all the assays were successfully performed. Multiple gene mutation and expression profiling of cancers has become a research priority and is expected to lead to personalized medicine for patients with many types of cancers. Many studies have reported mutation profiles in HCC and have identified approximately 30-40 mutations per tumor [18-20]. *TP53* and *CTNNB1*, which encodes β-catenin, are frequently mutated in HCC. On the other hand, few novel driver oncogene mutations have not been clarified in HCC. In the present study, we analyzed the mutation profile using targeted sequencing. Therefore, the numbers of detected mutations were limited. However, the mutations of these genes were frequently detected, suggesting that both Wnt and TP53 signaling are frequently altered in HCC.

In our study, we hypothesized that the oncogenic mutational burden could be a possible predictive biomarker for the efficacy of sorafenib in patients with HCC. We tried to detect any difference in the number of mutations in several oncogenes (*NRAS, PTPN11, CSF1R, ERBB2, FGFR2, FLT3, GNAQ, KRAS,* and *PIK3CA*) and found a significant difference in the number of oncogene mutations between the non-PD and PD groups. This result suggests that the oncogene mutational burden in the tumor might be associated with the clinical response to sorafenib, although a prospective study is necessary for confirmation.

In the RNA sequencing experiment, the expressions of approximately 50 genes were successfully measured and analyzed in all the biopsy samples, suggesting that FFPE biopsy samples of HCC are suitable for RNA sequencing. The increased gene expressions of *TGFa* and *PECAM1*, which encodes CD31, were observed in non-PD group. *PECAM1* is expressed in endothelial cells and is a famous vascular marker [21, 22]. A significantly high *PECAM1* expression level was observed in the non-PD group, suggesting that the vascular density of the tumor might be associated with the response to sorafenib. However, a significant difference in the PFS was not observed between the high and low *TGFa* groups or between the high and low *PECAM1* groups. On the other hand, the expression level of *NRG1* was significantly correlated with the PFS, although there was no difference in *NRG1* expression between the non-PD and the PD groups. NRG1 (also known as heregulin) is a ligand for HER3 (human epidermal growth factor receptor 3), and NRG1 stimulates HER2/HER3 heterodimerization, leading to the activation of HER2 signaling [23]. The EGFR antibody cetuximab is an effective clinical therapy for patients with colorectal, head and neck, and other solid tumors. We previously reported that the activation of ERBB2 signaling through the upregulation of NRG1 leads to cetuximab resistance [24]. The increased expression of NRG1 increases the autocrine signaling of HER2/HER3 in tumors. Thus, the activation of a bypass pathway mediated by NRG1 may lead to sorafenib resistance.

We previously reported that HCC tumors with *FGF3/FGF4* gene amplification responded to sorafenib [11]. In the presently reported series, we did not examine *FGF3/FGF4* amplification directly because of the limited sample sizes. However, we roughly screened the gene expressions of *FGF3* and *FGF4* using RNA sequencing and did not observe any increase in the samples (Supplementary Table S3). We considered these candidate genes related to the sorafenib response to be independent markers of *FGF3/FGF4* gene amplification.

In this study, we selected *TGFa, PECAM1*, and *NRG1* as predictive gene expression markers. Is one gene better than the others? In the present cohort, the PFS of the non-PD group was significantly longer than that of the PD group (106 days *vs*. 70 days, *P* = 0.0051, log-rank test). Response and PFS are considered to be good surrogates for evaluating the efficacy of sorafenib treatment. In this study, we focused on unresectable HCC treated with sorafenib. In this population, response is a possible surrogate for overall survival, reportedly [25, 26]. In this clinical study, overall survival was largely affected by post-sorafenib treatment including TACE, e.g. because a post-treatment protocol has not been prescribed. Therefore, the significance of these three markers should be confirmed in further studies.

In conclusion, we have demonstrated that 1) FFPE biopsy samples of HCC can be used for targeted DNA and RNA sequencing, 2) the tumor mutational burden is a candidate predictor of sorafenib effectiveness, and 3) the tumor gene expressions of *NRG1, TGFa*, and *PECAM1* are candidate markers for the prediction of sorafenib effectiveness. Further confirmatory studies will help to establish the utility of this biomarker profile for HCC.

## MATERIALS AND METHODS

### Patients and samples

Tumor specimens were obtained from a total of 46 HCC patients who chose to receive sorafenib at three hospitals in the Japanese Red Cross Liver Study Group between 2009 and 2013. All the patients received sorafenib (800 mg/body/day or 400 mg/body/day in patients with some risk factors [7]), and their responses were evaluated using the RECIST criteria [27]. The response to sorafenib was evaluated every 4–8 weeks by dynamic CT or MRI. This study was approved by the ethics committee of each institution. All the patients enrolled in the study provided written informed consent for the use of tumor tissues. The characteristics of the patients, including the efficacy results, are shown in Table 1.

### DNA and RNA extraction

The collected FFPE specimens underwent a histological review, and only those containing sufficient tumor cells as revealed by hematoxylin-eosin staining were subjected to nucleic acid extraction. DNA and RNA were purified using an Allprep DNA/RNA FFPE kit (Qiagen, Valencia, CA) according to the manufacturer's instructions. The quality and quantity of the DNA/RNA were verified using the NanoDrop 2000 device (Thermo Scientific Wilmington, DE), the PicoGreen dsDNA assay kit (Life Technologies, Foster City, CA), and the RiboGreen RNA assay kit (Life Technologies). The extracted DNA/RNA was stored at −80°C until the analysis.

### DNA sequencing

We used 10 ng of DNA for the multiplex PCR amplification using the Ion AmpliSeq Library kit 2.0 (Life Technologies) and the Ion AmpliSeq Cancer Hotspot Panel v2 (Life Technologies) according to the manufacturer's instructions. The genes for DNA sequencing are listed in Supplementary Table S5. The Ion Xpress Barcode Adapters (Life Technologies) were ligated into the PCR products and purified with Agencourt AMPure XP beads (Beckman Coulter, Brea, CA). The purified libraries were then pooled and sequenced on an Ion Torrent PGM device (Life Technologies) using the Ion PGM 200 Sequencing kit v2 (Life Technologies) and the Ion 318 v2 Chip kit (Life Technologies).

DNA sequencing data were accessed through the Torrent Suite v.4.0 software program. Reads were aligned against the hg19 human reference genome, and variants were called using the variant caller ver 4.0. Raw variant calls were filtered out using the following annotations: homozygous and heterozygous variants, quality score of < 100, depth of coverage < 19. Germline mutations were excluded using the Human Genetic Variation Database (http://www.genome.med.kyoto-u.ac.jp/SnpDB) [28].

### RNA sequencing

For RNA sequencing, PCR primers were designed using the Ion AmpliSeq Designer (Life Technologies). The genes for RNA sequencing are listed in Supplementary Table S5. Fifty target genes, consisting of receptor tyrosine kinase and its ligands, were selected for RNA sequencing. The Ion AmpliSeq RNA Library Kit (Life Technologies) was used to construct the RNA library according to the manufacturer's instructions. Briefly, 10 ng of total RNA were reverse transcribed with the SuperScript III enzyme, followed by PCR amplification. The Ion Xpress Barcode adapters (Life Technologies) were ligated into the PCR products and purified with Agencourt AMPure XP beads (Beckman Coulter). Purified libraries were then pooled and sequenced on an Ion Torrent PGM device using the Ion PGM 200 Sequencing kit v2 and the Ion 318 v2 Chip kit. Relative expression (raw read number/total read number) of each gene was calculated for the normalization.

### Statistical analysis

A Chi-squared test was used to compare the number of mutations and the treatment response. A non-parametric statistical method (Mann-Whitney U-test) was used for comparisons between the expressions of various genes and treatment response. The Kaplan-Meier method and the log-rank test were used to analyze survival. All the statistical analyses were performed using JMP software (ver 10, SAS Institute). A *P* value of < 0.05 was considered statistically significant.

## SUPPLEMENTARY TABLES











## References

[1] Kim do Y, Han KH (2012). Epidemiology and surveillance of hepatocellular carcinoma. Liver Cancer.

[2] Kudo M (2014). Prediction of hepatocellular carcinoma incidence risk by ultrasound elastography. Liver Cancer.

[3] Revill K, Wang T, Lachenmayer A, Kojima K, Harrington A, Li J, Hoshida Y, Llovet JM, Powers S (2013). Genome-wide methylation analysis and epigenetic unmasking identify tumor suppressor genes in hepatocellular carcinoma. Gastroenterology.

[4] Wong N, Lai P, Pang E, Fung LF, Sheng Z, Wong V, Wang W, Hayashi Y, Perlman E, Yuna S, Lau JW, Johnson PJ (2000). Genomic aberrations in human hepatocellular carcinomas of differing etiologies. Clin Cancer Res.

[5] Cheng AL, Kang YK, Chen Z, Tsao CJ, Qin S, Kim JS, Luo R, Feng J, Ye S, Yang TS, Xu J, Sun Y, Liang H (2009). Efficacy and safety of sorafenib in patients in the Asia-Pacific region with advanced hepatocellular carcinoma: a phase III randomised, double-blind, placebo-controlled trial. Lancet Oncol.

[6] Llovet JM, Ricci S, Mazzaferro V, Hilgard P, Gane E, Blanc JF, de Oliveira AC, Santoro A, Raoul JL, Forner A, Schwartz M, Porta C, Zeuzem S (2008). Sorafenib in advanced hepatocellular carcinoma. N Engl J Med.

[7] Takeda H, Nishikawa H, Osaki Y, Tsuchiya K, Joko K, Ogawa C, Taniguchi H, Orito E, Uchida Y, Izumi N, Japanese Red Cross Liver Study G (2015). Clinical features associated with radiological response to sorafenib in unresectable hepatocellular carcinoma: a large multicenter study in Japan. Liver Int.

[8] Inuzuka T, Nishikawa H, Sekikawa A, Takeda H, Henmi S, Sakamoto A, Saito S, Kita R, Kimura T, Osaki Y, Kudo M (2011). Complete response of advanced hepatocellular carcinoma with multiple lung metastases treated with sorafenib: a case report. Oncology.

[9] Nakazawa T, Hidaka H, Shibuya A, Koizumi W (2010). Rapid regression of advanced hepatocellular carcinoma associated with elevation of des-gamma-carboxy prothrombin after short-term treatment with sorafenib - a report of two cases. Case Rep Oncol.

[10] So BJ, Bekaii-Saab T, Bloomston MA, Patel T (2008). Complete clinical response of metastatic hepatocellular carcinoma to sorafenib in a patient with hemochromatosis: a case report. J Hematol Oncol.

[11] Arao T, Ueshima K, Matsumoto K, Nagai T, Kimura H, Hagiwara S, Sakurai T, Haji S, Kanazawa A, Hidaka H, Iso Y, Kubota K, Shimada M (2013). FGF3/FGF4 amplification and multiple lung metastases in responders to sorafenib in hepatocellular carcinoma. Hepatology.

[12] Fujimoto A, Totoki Y, Abe T, Boroevich KA, Hosoda F, Nguyen HH, Aoki M, Hosono N, Kubo M, Miya F, Arai Y, Takahashi H, Shirakihara T (2012). Whole-genome sequencing of liver cancers identifies etiological influences on mutation patterns and recurrent mutations in chromatin regulators. Nat Genet.

[13] Villanueva A, Llovet JM (2014). Liver cancer in 2013: Mutational landscape of HCC—the end of the beginning. Nat Rev Clin Oncol.

[14] Lievre A, Bachet JB, Boige V, Cayre A, Le Corre D, Buc E, Ychou M, Bouche O, Landi B, Louvet C, Andre T, Bibeau F, Diebold MD (2008). KRAS mutations as an independent prognostic factor in patients with advanced colorectal cancer treated with cetuximab. J Clin Oncol.

[15] Okamoto I, Sakai K, Morita S, Yoshioka H, Kaneda H, Takeda K, Hirashima T, Kogure Y, Kimura T, Takahashi T, Atagi S, Seto T, Sawa T (2014). Multiplex genomic profiling of non-small cell lung cancers from the LETS phase III trial of first-line S-1/carboplatin versus paclitaxel/carboplatin: results of a West Japan Oncology Group study. Oncotarget.

[16] Sakai K, Kazama S, Nagai Y, Murono K, Tanaka T, Ishihara S, Sunami E, Tomida S, Nishio K, Watanabe T (2014). Chemoradiation provides a physiological selective pressure that increases the expansion of aberrant TP53 tumor variants in residual rectal cancerous regions. Oncotarget.

[17] Oshlack A, Robinson MD, Young MD (2010). From RNA-seq reads to differential expression results. Genome Biol.

[18] Cleary SP, Jeck WR, Zhao X, Chen K, Selitsky SR, Savich GL, Tan TX, Wu MC, Getz G, Lawrence MS, Parker JS, Li J, Powers S (2013). Identification of driver genes in hepatocellular carcinoma by exome sequencing. Hepatology.

[19] Guichard C, Amaddeo G, Imbeaud S, Ladeiro Y, Pelletier L, Maad IB, Calderaro J, Bioulac-Sage P, Letexier M, Degos F, Clement B, Balabaud C, Chevet E (2012). Integrated analysis of somatic mutations and focal copy-number changes identifies key genes and pathways in hepatocellular carcinoma. Nat Genet.

[20] Kan Z, Zheng H, Liu X, Li S, Barber TD, Gong Z, Gao H, Hao K, Willard MD, Xu J, Hauptschein R, Rejto PA, Fernandez J (2013). Whole-genome sequencing identifies recurrent mutations in hepatocellular carcinoma. Genome Res.

[21] Arao T, Matsumoto K, Furuta K, Kudo K, Kaneda H, Nagai T, Sakai K, Fujita Y, Tamura D, Aomatsu K, Koizumi F, Nishio K (2011). Acquired drug resistance to vascular endothelial growth factor receptor 2 tyrosine kinase inhibitor in human vascular endothelial cells. Anticancer Res.

[22] Kaneda H, Arao T, Tanaka K, Tamura D, Aomatsu K, Kudo K, Sakai K, De Velasco MA, Matsumoto K, Fujita Y, Yamada Y, Tsurutani J, Okamoto I (2010). FOXQ1 is overexpressed in colorectal cancer and enhances tumorigenicity and tumor growth. Cancer Res.

[23] Sakai K, Yokote H, Murakami-Murofushi K, Tamura T, Saijo N, Nishio K (2007). Pertuzumab, a novel HER dimerization inhibitor, inhibits the growth of human lung cancer cells mediated by the HER3 signaling pathway. Cancer Sci.

[24] Yonesaka K, Zejnullahu K, Okamoto I, Satoh T, Cappuzzo F, Souglakos J, Ercan D, Rogers A, Roncalli M, Takeda M, Fujisaka Y, Philips J, Shimizu T (2011). Activation of ERBB2 signaling causes resistance to the EGFR-directed therapeutic antibody cetuximab. Sci Transl Med.

[25] Edeline J, Boucher E, Rolland Y, Vauléon E, Pracht M, Perrin C, Le Roux C, Raoul JL (2012). Comparison of tumor response by Response Evaluation Criteria in Solid Tumors (RECIST) and modified RECIST in patients treated with sorafenib for hepatocellular carcinoma. Cancer.

[26] Ronot M, Bouattour M, Wassermann J, Bruno O, Dreyer C, Larroque B, Castera L, Vilgrain V, Belghiti J, Raymond E, Faivre S (2014). Alternative Response Criteria (Choi, European association for the study of the liver, and modified Response Evaluation Criteria in Solid Tumors [RECIST]) Versus RECIST 1.1 in patients with advanced hepatocellular carcinoma treated with sorafenib. Oncologist.

[27] Sato Y, Watanabe H, Sone M, Onaya H, Sakamoto N, Osuga K, Takahashi M, Arai Y, Japan Interventional Radiology in Oncology Study Group J (2013). Tumor response evaluation criteria for HCC (hepatocellular carcinoma) treated using TACE (transcatheter arterial chemoembolization): RECIST (response evaluation criteria in solid tumors) version 1.1 and mRECIST (modified RECIST): JIVROSG-0602. Ups J Med Sci.

[28] Narahara M, Higasa K, Nakamura S, Tabara Y, Kawaguchi T, Ishii M, Matsubara K, Matsuda F, Yamada R (2014). Large-scale East-Asian eQTL mapping reveals novel candidate genes for LD mapping and the genomic landscape of transcriptional effects of sequence variants. PLoS One.

